# The Effect of Intermittent Fasting on Salivary Inflammatory Cytokines and Dopamine Levels

**DOI:** 10.1055/s-0045-1802345

**Published:** 2025-03-12

**Authors:** Elyazia Fayyad, Zumurd Al Anoud, Abdulkader Habra, Hussein Al Meshal, Aghila Rani K.G, Roba Saqan, Zuha Rizvi, Manal Awad, Natheer Al-Rawi

**Affiliations:** 1 College of Dental Medicine, University of Sharjah, Sharjah, United Arab Emirates; 2 Research Institute for Medical and Health Sciences, University of Sharjah, Sharjah, United Arab Emirates; 3 Department of Orthodontics, Preventive & Community Dentistry, College of Dental Medicine, University of Sharjah, Sharjah, United Arab Emirates; 4 Department of Oral & Craniofacial Health Sciences, College of Dental Medicine, University of Sharjah, Sharjah, United Arab Emirates; 5 Department of Oral Diagnosis, College of Dentistry, Mashreq University, Baghdad, Iraq

**Keywords:** Ramadan fasting, dopamine, saliva, interleukins, ELISA

## Abstract

**Objectives:**

The current study elucidates potential alterations in inflammatory cytokines and dopamine (DA) levels in saliva following a 21-day fasting regimen during Ramadan and explores their associations with mental health parameters.

**Materials and Methods:**

Forty-four healthy young university students (22 females and 22 males; median age 22 (2) and body mass index 23.40 (6.23) were enrolled, and saliva samples were collected before and after fasting. Cytokine and DA levels were quantified by ELISA and LEGENDplex Human Inflammation Panel, respectively. Participants also completed the Patient Health Questionnaire-9 and Depression Anxiety Stress Scales questionnaires to assess mental health.

**Statistical Analysis:**

Data analysis was performed using SPSS. Differences between pre- and postfasting were tested using Wilcoxon's signed-rank test. Mann–Whitney's *U* test determined disparities in DA and cytokine levels across sex. Simple and multiple linear regression analyses were performed to identify the factors influencing the change in DA. Correlation analysis was employed to explore the relationships between the changes in DA and cytokine levels before and after fasting.

**Results:**

A significant increase in inflammatory cytokines such as interleukin (IL)-1β, interferon-α2, tumor necrosis factor-α, IL-23, IL-33, and IL-8 was observed after fasting. Anti-inflammatory cytokine, IL-10, levels remained unchanged. Females had significantly higher levels of proinflammatory cytokines before fasting compared with males, but no significant gender differences were observed after fasting. The current study also showed a significant decrease in DA levels after fasting; however, no significant difference in DA levels across genders was noted. Self-reported mental health status did not significantly change before and after fasting. Multiple linear regression analyses did not suggest potential associations between these variables and changes in DA levels before and after fasting. However, correlation analysis indicated that the change in inflammatory cytokine levels was inversely related to changes in DA levels.

**Conclusion:**

Fasting during Ramadan significantly increased salivary cytokine levels and decreased DA levels, indicating potential relationships between immune factors and mental well-being. The findings highlight the complex interplay between inflammation, immunity, and DA regulation during fasting. Further research is warranted to elucidate the potential long-term effects of these associations and their implications for mental health and well-being.

## Introduction

Intermittent fasting has garnered significant attention in recent years due to its potential impact on various physiological and metabolic processes, including alterations in neurotransmitter levels and inflammatory responses. 1 Among the neurotransmitters affected by intermittent fasting, dopamine (DA) holds particular significance, given its crucial role in regulating mood and motivation. 2 Abnormalities in DA signaling have been implicated in mood disorders such as depression, bipolar disorder, and addiction. 2 Understanding the changes in DA levels induced by fasting could provide valuable insights into its potential therapeutic effects on mood-related conditions. 2

Intermittent fasting has been associated with numerous health benefits beyond mood regulation. Prior studies demonstrated improvements in insulin sensitivity, lipid metabolism, gut microbiota health, and reduced inflammation and blood pressure among individuals practicing intermittent fasting. 3 The potential anti-inflammatory effects of intermittent fasting, particularly during practices such as Ramadan fasting, have shown promise in alleviating inflammatory conditions such as rheumatoid arthritis. 4 However, a recent review reported that intermittent fasting has little or no effect on key inflammatory markers. 5

Inflammatory cytokines play an important role in maintaining neuronal integrity and managing neurotransmitter systems including DA release in the brain. There is mounting interest regarding their role in the onset of several behavioral alterations, with the excess presence of inflammatory cytokines regarded as the primary cause for alterations in DA secretion. 6 However, the impact of intermittent fasting on inflammatory cytokine secretion and its subsequent effects on DA levels is largely elusive, especially considering that the levels of these inflammatory cytokines are transient. Regarding the impact of intermittent fasting on inflammatory cytokine, some studies suggest significant reductions in proinflammatory markers, 7 8 while others report limited effects. 5 Discrepancies in findings may stem from variations in fasting protocols, sample sizes, study samples, and design, highlighting the need for further research, particularly well-powered randomized controlled trials, to elucidate the mechanisms underlying these effects.

Saliva, as a biological fluid, offers a noninvasive and easily accessible means for elucidating cytokine and neurotransmitter levels. 9 The close relationship between the salivary glands and the nervous system makes the secretions from these glands a valuable source of biomarkers, including normal and pathological conditions of the nervous system. 10 The use of salivary biomarkers presents several advantages, including cost-effectiveness, ease of sample collection, and repeatability, making it a valuable alternative method. 11 However, there is limited research identifying transient changes in salivary biomarkers influenced by intermittent fasting. Comparative studies and evaluations of the clinical relevance of these biomarkers are necessary to enhance the use of saliva as a noninvasive diagnostic tool. In this context, the present study aims to comprehensively investigate changes in salivary levels of inflammatory cytokines and DA before and after Ramadan fasting among young university students. By employing a noninvasive and objective approach to sample collection, this study seeks to address existing gaps in the literature regarding the detection of salivary biomarkers and their potential alterations following intermittent fasting. Furthermore, the inclusion of dental students as a study population offers a unique opportunity to explore the effects of fasting on individuals undergoing academic stress, which may impact both their mental and physical well-being. This study is particularly novel in its use of saliva samples, as prior studies have predominantly utilized blood samples or animal models, thus providing new insights into young students' physiological and psychological responses to fasting.

## Materials and Methods

### Population and Study Design

The current observational study collected data at a single point of time using a cross-sectional quantitative methodology. Forty-four young healthy fasting participants (22 females and 22 men) who were all students enrolled in the College of Dental Medicine at the University of Sharjah were recruited in the study. Informed written consent was obtained from all the study participants, and the design and nature of the study were also detailed. The participants were requested to complete the Patient Health Questionnaire (PHQ)-9 and Depression Anxiety Stress Scales questionnaires during saliva sample collection (both before fasting and at the end of fasting). The study procedure was approved by the University of Sharjah's Research Ethics Committee (REC-22-03-21-03-S) by national and international norms and the Declaration of Helsinki.

Inclusion criteria were the following: young participants in good oral health who fasted for 21 consecutive days for 13 hours (from 5 a.m. to 6:30 p.m.). Clinical evaluation procedures included an absence of calculus, active caries, tooth brushing twice per day, and maintaining good oral hygiene. We excluded subjects having a history of drug abuse, eating disorders, malnutrition, diabetes, hypertension, renal impairment, and neurological or mental diseases. Also, smokers and subjects taking any drugs that might influence salivary flow were excluded from the study.

### Saliva Collection

This observational study aimed to quantify cytokine and DA levels in saliva before and after fasting for 21 consecutive days for 13 hours (from 5 a.m. to 6:30 p.m.). Saliva samples were collected twice, the first sample was collected a day before fasting, and the second sample at 3 weeks following the fasting period. The collection time of both samples was between 11 a.m. and 1 p.m. Passive drooling was followed for saliva collection. Participants were instructed to be seated with heads tilted forward, allowing saliva to pool naturally in the mouth and 5 mL of unstimulated whole saliva was collected from each participant for 10 minutes in sterile test tubes, labeled with a unique identification number. The samples were transported immediately to the laboratory in ice storage boxes. The saliva samples were further centrifuged at 2,500 rpm for 5 minutes to reduce multiple freeze-thaw cycles and remove cell debris and mucus contamination, aliquoted and stored at −80°C freezer until the required number of samples ( *n* = 40) was achieved. On the day of the multiplex and ELISA analysis, saliva samples were further thawed in ice, centrifuged at 10,000 g for 10 minutes at 4°C, and supernatants were collected for use in the study.

### Cytometric Bead Array for the Estimation of Cytokine Levels in the Saliva Samples

Cytometric bead array was performed to determine the levels of the cytokines in the saliva samples using the LEGENDplex Human Inflammation Panel 1 (13-plex) (Cat. no.: 740809; BioLegend, San Diego, California, United States). The kit quantifies the level of cytokines including interleukin (IL)-1β, interferon (IFN)-α2, IFN-γ, tumor necrosis factor (TNF)-α, monocyte chemoattractant protein (MCP)-1, IL-6, C-X-C motif chemokine ligand 8 (IL-8), IL-10, IL-12p70, IL-17A, IL-18, IL-23, and IL-33 in the saliva samples. The standard graphs and the experimental protocol were performed according to the manufacturer's guidelines. Briefly, 25 μL of the saliva sample mixed with equal volumes of assay buffer was incubated with the 25 μL microbeads for 2 hours in the dark with mild rotation (800 rpm). The samples were washed with 350 μL 1 *x* wash buffer and centrifuged at 2,000 rpm for 5 minutes. The supernatant was carefully removed and the pellet was resuspended in 25 μL of the detection antibodies and further incubated for 1 hour at room temperature (with mild shaking at 800 rpm). After incubation, 25 μL of the streptavidin–phycoerythrin was introduced subsequently to the standards and test samples and incubated further for 30 minutes. The samples were then washed with 350 μL wash buffer and centrifuged at 2,000 rpm for 5 minutes. The supernatant was carefully removed and the pellet was resuspended in 200 μL wash buffer. The samples were then acquired on a flow cytometer (BD Cytoflex, BD Biosciences, United States) and analysis was performed by using LEGENDplex Data Analysis Software (BioLegend).

### ELISA Analysis for Estimation of Salivary Dopamine Levels

Detection of DA levels in the saliva samples was performed using a human DA ELISA kit (Cat. no.: E-EL-0046; Elabscience, United States). Briefly, 50 µL of saliva samples were loaded into ELISA plates along with 50 µL of biotinylated detection antibody, and the assay was performed according to the manufacturer's instructions. The samples were incubated for 45 minutes at 37°C followed by washing with 1 *x* wash buffer three times; 100 µL of horseradish peroxidase conjugate was further added to each well and incubated for 30 minutes at 37°C. The ELISA plate was then washed five times and 90 µL of substrate reagent was added and further incubated for 15 minutes at 37°C. At the end of the incubation period, 50 µL of STOP solution was added and absorbance read at 450 nm. A standard plot was prepared and the concentration of DA in the study samples was extrapolated and interpreted in pg/mL.

### Statistical Analysis

Data analysis was performed using SPSS (Statistical Package for the Social Sciences) (Version 22, IBM Corp., United States). The descriptive statistics for continuous data were reported as median and interquartile range (IQR) and for categorical data as frequencies and percentages. Differences between pre- and postfasting were tested using Wilcoxon's signed-rank test, depending on data normality. The Mann–Whitney's *U* test was utilized to determine disparities in DA and cytokine levels across sex. Simple and multiple linear regression analyses were performed to identify the factors influencing the change in DA before and after fasting. Variables in the simple regression model with a *p* -value less than 0.2 were included in the multiple regression model. Additionally, correlation analysis was employed to explore the relationships between the change in DA level before and after fasting and the change in cytokine level before and after fasting. Correlation analysis was also performed between the change in DA level before and after fasting and the change in mental health parameter scores before and after fasting including stress, anxiety, depression, and PHQ. Bar graphs were generated using Graph Pad PRISM (version 9.1.1). A *p* -value of less than 0.05 was considered statistically significant.

## Results

The study participants ( *n* = 44) were United Arab Emirates residents and young adults of age between 18 and 24 years. The median age of enrolled participants was 22 (2) and their median body mass index (BMI) was 23.40 (6.23). No difference in BMI was observed among the male and female participants. The participants completed a multicomponent questionnaire related to their sociodemographic and mental health status including anxiety, depression, and severity of depression.

### Fasting Enhanced Salivary Proinflammatory Cytokine Levels

The findings of Wilcoxon's signed-rank test conducted to investigate the difference in salivary cytokine levels before and after fasting are presented in Table 1 . Interestingly, a statistically significant increase in certain key proinflammatory cytokines such as IL-1β, TNF-α, IL-23, IL-33, and IL-8 was noted. Additionally, IFN-α2 levels were also significantly enhanced after fasting. There was significant increase in IL-1β scores from before fasting (median [IQR]: 396.16 [721.00] vs. after fasting 569.45 [882.00]), with a *p* = 0.013. Similar findings were observed in the following cytokines (before fasting vs. after fasting), such as IFN-α2: 0.25 (0) versus 0.38 (0), *p* = 0.010; TNF-α: 10.24 (18) versus 18.61 (18.00), *p* = 0.040; and IL-8: 1,373.34 (1,425.00) vs. 1,966.21 (1,636.00), *p* = 0.010. Furthermore, a highly significant increase ( *p* < 0.001) was observed in IL-23 and IL-33 levels (before vs. after fasting) with the median (IQR): 8.72 (9.00) versus 13.88 (10.00) and 3.20 (6.00) versus 9,10 (11.00), respectively.

**Table 1:** Impact of fasting on salivary levels of pro- and anti-inflammatory cytokines

Variables	Median (IQR)		*p* -Value
	Before fasting	After fasting	
IFN-γ	2.52 (6.00)	3.74 (5.00)	0.253
IL-1β	396.16 (721.00)	569.45 (882.00)	0.013 a
IFN-α	0.25 (0)	0.38 (0)	0.010 a
TNF-α	10.24 (18.00)	18.61 (18.00)	0.040 a
MCP-1	449.65 (459.00)	491.52 (486.00)	0.779
IL-23	8.72 (9.00)	13.88 (10.00)	<0.001 a
IL-33	3.20 (6.00)	9.10 (11.00)	<0.001 a
IL-18	754.62 (1,080.00)	1,005.00 (1,295.00)	0.084
IL-17	0.10 (0)	0.10 (0)	0.987
IL-12	1.37 (6.00)	2.31 (3.00)	0.359
IL-10	3.56 (7.00)	4.63 (8.00)	0.064
IL-8	1,373.34 (1,425.00)	1,966.21 (1,636.00)	0.010 a
IL-6	8.35 (10.00)	10.06 (12.00)	0.076

Abbreviations: IFN, interferon; IL, interleukin; IQR, interquartile range; MCP, monocyte chemoattractant protein; TNF, tumor necrosis factor.

a *p* -Value is significant at 0.05.

**Fig. 1:**
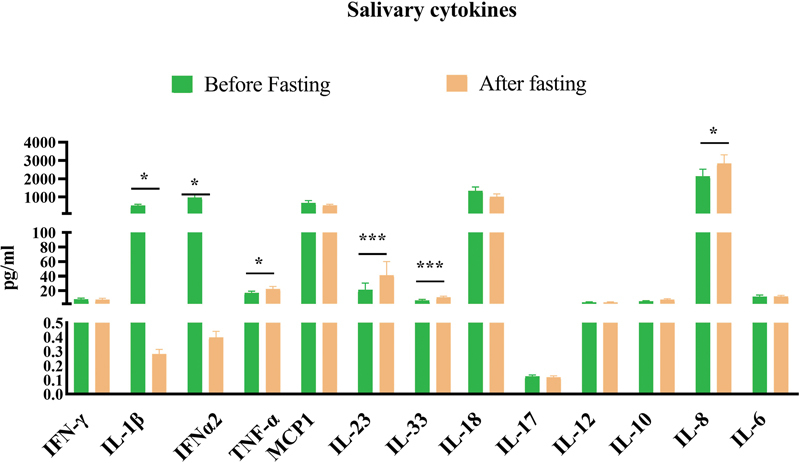
Flow cytometry-based analysis of human salivary cytokines. The quantification and comparative analysis of cytokines in the salivary samples was assessed by a bead-based immunoassay on a flow cytometer using LEGENDplex Human Inflammation Panel (13-plex). ^*^ *p* < 0.05 and ^***^ *p* < 0.001. IFN, interferon; IL, interleukin; MCP1, monocyte chemoattractant protein 1; TNF-α, tumor necrosis factor α.

On the other hand, there was no significant difference in the expression of the anti-inflammatory cytokine IL-10 before and after fasting. This indicates that fasting had a statistically significant impact on increasing salivary proinflammatory cytokine levels ( Table 1 and Fig. 1 ).

We observed a statistically significant difference in the median scores of nine proinflammatory cytokines in saliva samples collected before fasting between females and males. Overall, females had significantly higher levels of cytokines compared with males before fasting. These cytokines include IFN-α (males vs. females): 0.13 (0) versus 0.33 (0) before fasting, *p* < 0.001; TNF-α: 9.12 (13) versus 18.64 (21), *p* = 0.046; MCP-1: 331.74 (480) versus 578.71 (763), *p* = 0.031; IL-23: 7.63 (8) versus 11.85 (16), *p* = 0.012; IL-33: 1.70 (5) versus 5.30 (15), *p* = 0.004; IL-12: 0.77 (1) versus 2.29 (7), *p* = 0.012; IL-10: 1.63 (4) versus 4.68 (12), *p* = 0.005; IL-8: 1,171.36 (1,616) versus 1,544.33 (1,235), *p* = 0.037; and IL-6: 4.83 (10) versus 9.57 (11), *p* = 0.039. However, there was no statistically significant difference in cytokine levels among males and females after fasting ( Supplementary Table S1 [available in the online version only]).

### Fasting Alters Salivary Dopamine Levels but Not Mental Health Parameters

**Fig. 2:**
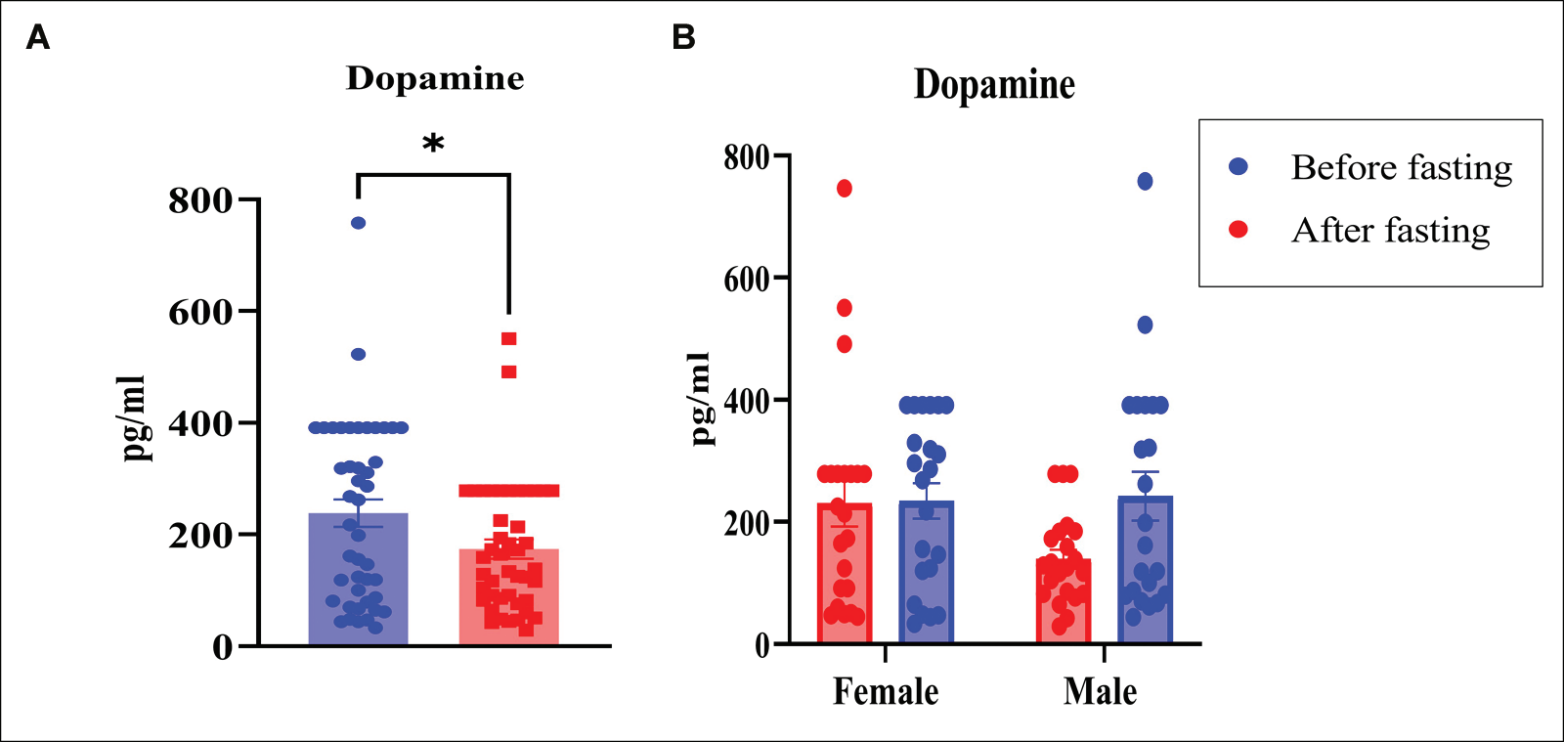
Comparison of median salivary dopamine scores before and after fasting. ( **A** ) The median scores of salivary dopamine altered significantly before and after fasting among the study participants by the Wilcoxon's signed-rank test. ( **B** ) The median scores of salivary dopamine were comparable across genders before and after fasting by the Mann–Whitney's *U* test.

The findings presented in Table 2 indicate the results of the Wilcoxon's signed-rank test conducted to investigate the difference in DA, and mental health parameters among the study participants before and after fasting. The results indicate a significant decrease in DA levels, median (IQR): 217.40 (309.88) before fasting versus 133.60 (196.41) after fasting with a *p* -value of 0.016 ( Table 2 and Fig. 2A ). However, no significant difference in DA levels across genders was noted ( Fig. 2B ). Similarly, no significant difference was detected in the mental health status of the study participants before and after fasting ( Table 2 ).

**Table 2:** Impact of fasting on salivary DA and mental health

	Median (IQR)		*p* -Value
	Before fasting	After fasting	
DA	217.40 (309.88)	133.60 (196.41)	0.016 a
Stress	3.00 (3)	3.00 (3)	0.168
Anxiety	3.00 (3.75)	2.50 (3)	0.286
Depression	4.00 (3)	3.00 (2)	0.860
PHQ	9.00 (6.75)	8.00 (4)	0.720

Abbreviations: DA, dopamine; IQR, interquartile range; PHQ, Patient Health Questionnaire.

a *p* -Value is significant at 0.05.

### Factors Influencing Changes in Dopamine Levels (before and after Fasting)

Table 3 shows the results of the multiple regression analysis performed to examine the influence of the change in specific cytokines and the PHQ scores before and after fasting on the change in the DA level (before and after fasting). It was observed that cytokines such as IFN-α2 ( *p* = 0.021), TNF-α ( *p* = 0.017), MCP-1 ( *p* = 0.048), IL-33 ( *p* = 0.030), and IL-10 ( *p* = 0.010) showed a significant effect on the change in DA levels by simple linear regression analysis ( Table 3 ). For multiple regression analysis, 11 variables were included including the cytokines with a *p* -value less than 0.2 in simple linear regression. However, the study revealed an insignificant relationship between these variables and the change in DA levels (before and after fasting).

**Table 3:** Multiple linear regression

Δ DA	Simple linear regression			Multiple linear regression		
	Β	95% CI	*p* -Value	Adj β	95% CI	*p* -Value
Δ IL-1β	−0.05	−0.11 to 0	0.057	0.04	−0.07 to 0.15	0.435
Δ IFN-α2	−175.14	−323.01 to −27.28	0.021 a	51.51	−221.34 to 324.36	0.703
Δ TNF-α	−3.89	−7.05 to −0.72	0.017 a	−4.14	−10.79 to 2.51	0.213
Δ MCP-1	−0.06	−0.13 to 0	0.048 a	−0.09	−0.21 to 0.04	0.166
Δ IL-23	−0.61	−1.40 to 0.18	0.126	0.05	−1.01 to 1.11	0.925
Δ IL-33	−4.52	−8.56 to −0.47	0.030 a	−0.45	−7.93 to 7.03	0.903
Δ IL-17	−424.69	−979.49 to 130.10	0.130	−505.09	−1,129.50 to 119.32	0.109
Δ IL-12	−8.66	−20.31 to 3	0.141	3.30	−14.93 to 21.53	0.715
Δ IL-10	−7.61	−13.29 to −1.92	0.010 a	−8.12	−17.74 to 1.51	0.095
Δ IL-6	−1.76	−4.76 to 1.23	0.242	3.45	−1.92 to 8.82	0.199
Δ PHQ	12.02	−5.52 to 29.57	0.174	14.08	−4.24 to 32.40	0.127

Abbreviations: Δ, difference before and after fasting; Adj β, adjusted β; CI, confidence interval; DA, dopamine; IFN, interferon; IL, interleukin; MCP, monocyte chemoattractant protein; PHQ, Patient Health Questionnaire; TNF, tumor necrosis factor; β, regression coefficient.

Note: Determinants of changes in DA levels before and after fasting.

a *p* -Value is significant at 0.05.

### Correlation Analysis

The results of the correlation analysis are given in Table 4 . The study revealed that the change in certain specific cytokine levels before and after fasting is significantly correlated with the change in DA levels (before and after fasting). Specifically, the findings showed a significant negative correlation between the change in DA levels and the change in levels of IFN-α ( *r* = − 0.346, *p* = 0.021); TNF-α ( *r* = − 0.357, *p* = 0.017); MCP-1 ( *r* = − 0.300, *p* = 0.048), IL-33 ( *r* = − 0.328, *p* = 0.030), and IL-10 ( *r* = − 0.385, *p* = 0.010) ( Table 4 ).

**Table 4:** Correlation between changes in cytokine and mental health scores and changes in DA levels before and after fasting

ΔDA		IFN-α	TNF-α	MCP-1	IL-23	IL-33	IL18	IL-17	IL-12	IL-10	IL-8	IL-6	Stress	Anxiety	Depression	PHQ
	r	−0.346 a	−0.357 a	−0.300 a	−0.234	−0.328 a	−0.090	−0.232	−0.225	−0.385 b	−0.090	−0.180	0.138	0.011	0.127	0.209
*p* -Value		0.021	0.017	0.048	0.126	0.030	0.562	0.130	0.141	0.010	0.562	0.242	0.371	0.943	0.412	0.174

Abbreviations: Δ, change in levels before and after fasting; DA, dopamine; IFN, interferon; IL, interleukin; MCP, monocyte chemoattractant protein; PHQ, Patient Health Questionnaire; r, correlation coefficient; TNF, tumor necrosis factor.

a *p* -Value is significant at 0.05.

b *p* -Value is significant at 0.01.

## Discussion

The innate immune system plays a central role in controlling behavior through changes in neurocircuitry and neurotransmitter systems in the brain. 12 This is partially due to the effect of inflammatory cytokines on monoamine neurotransmission. 13 DA is the key target of inflammatory cytokines, responsible for cytokine-induced behavioral changes. To the best of our knowledge, this is the first study that comprehensively assessed the effects of fasting on the levels of a panel of proinflammatory and anti-inflammatory cytokines in saliva before and after Ramadan and correlated its effects on DA release, mental health, and well-being of individuals.

The present study was conducted during Ramadan in United Arab Emirates among university students. The results demonstrate that fasting during Ramadan significantly altered salivary proinflammatory cytokine levels. We found a significant increase in the level of proinflammatory cytokines such as IL-1β, TNF-α, IL-23, IL-33, and IL-8, while IL-10 levels remain unchanged. The observed increase in proinflammatory cytokines suggests that fasting may induce an inflammatory response in the oral cavity, potentially as a physiological reaction to dietary changes. This is in contrast to findings from prior research. Tastemur et al reported that fasting during Ramadan decreased plasma levels of proinflammatory cytokines (TNF-α, IL-8), heat shock protein 70, and oxidative stress markers. 7 Similarly, a meta-analysis of 10 studies on individuals practicing fasting during Ramadan showed a marginal decrease in inflammatory biomarkers while fasting. 8 Yet, another study reported a decline in circulating proinflammatory cytokines, body fat, and leukocyte levels during Ramadan fasting indicating an attenuation of the body's inflammatory status. 8 The discrepancy in data may be due to differences in study methodologies, such as the use of blood and animal models and the median age of the participants. It is important to note that in the present study, all participants recruited were young university students. The alterations in energy provision and metabolic control linked to fasting might elicit the body's reaction to fluctuations in nutrient accessibility, 14 which could potentially result in elevated concentrations of inflammatory cytokines, as shown in our study.

Additionally, a baseline difference in cytokine levels between genders was noted, with females exhibiting higher levels before fasting. Such a disparity may be due to various biological factors, including hormonal differences, which are known to influence immune responses. 15 However, after fasting, the difference in cytokine levels between males and females is negligible suggesting that fasting may have normalized the variations in cytokine production and potentially mitigated the initial gender-based differences. The current study also showed that there was no significant change in the levels of the anti-inflammatory cytokine IL-10, indicating that the inflammatory response was not counterbalanced by anti-inflammatory mechanisms in the oral cavity.

Fasting alters reward-related behaviors and decreases baseline DA levels. 16 Also, several innate immune cytokines such as IFN-α is reported to produce high rates of behavioral disturbances, including depression and fatigue. 17 DA is involved in multiple behaviors, including feeding, and chronic fasting is known to affect DA neural circuits significantly due to a reduction in food intake and associated body weight. Previous research has also explored various aspects of DA regulation, its impact on metabolic programming, and immune responses. 18 Also, the impact of DA signaling on motivation, decision-making, and reward processes. 19 However, the effects of short-term changes in food intake, such as acute fasting, affecting DA circuits are not much explored.

Interestingly, in the current study, a significant decrease in salivary DA levels after fasting was noted. The change in DA levels, however, did not correspond to changes in mental health parameters such as anxiety, depression, and severity of depression during the short time frame of the study. This finding suggests that while fasting alters neurochemical markers such as DA, it does not significantly affect self-reported mental health status of the participants. The reduction in DA could be indicative of changes in reward- and stress-related pathways, which may be influenced by dietary intake and fasting. 16 The lack of change in mental health status despite changes in cytokine and DA levels could be attributed to the fact that the physiological stress of fasting does not immediately translate to perceivable changes in mental health, 20 given that the study was for a short period.

Further in the study, we observed that cytokines such as IFN-α2, TNF-α, MCP-1, IL-33, and IL-10 showed a significant effect on the change in DA levels by the simple linear regression analysis. However, when included in a multiple regression model, none of these cytokines significantly predicted the change in DA levels. This could also be attributed to the short duration of the fasting period, and long-term studies could provide more comprehensive insights into the collective effect of these cytokines on DA release. However, significant negative correlations between changes in DA levels and changes in levels of IFN-α2, TNF-α, MCP-1, IL-33, and IL-10 were observed. This inverse relationship implies that as levels of these cytokines increase, DA levels tend to decrease, highlighting a potential link between inflammatory processes and DA regulation.

The current study is limited owing to a single study sample, as saliva samples were collected exclusively from university students who are typically under stress. Additionally, our study was conducted on a sample of students enrolled in one institute, which may limit its generality to a broader population. Finally, the mental health was evaluated based on self-reported questionnaires.

## Conclusion

In conclusion, the current study demonstrates that fasting during Ramadan significantly alters salivary proinflammatory cytokine levels and DA release in youths. The mental health status of these young participants, however, remains unaffected in this short time frame of the study. The study highlights complex interactions between fasting, immune response, and neurochemical changes, warranting further research to explore the underlying mechanisms and long-term collective effects of the inflammatory cytokines on DA release and overall well-being. Correlations were identified between changes in DA levels and changes in levels of cytokines and chemokines such as IFN-α2, TNF-α, MCP-1, IL-33, and IL-10 before and after fasting. Overall, the potential predictive value of these associations as indicators of dopaminergic responses to fasting and the mechanisms underlying these associations are suggested based on our study findings.
