# Supplementary material for: The Effect of Intermittent Fasting on Salivary Inflammatory Cytokines and Dopamine Levels
Source: Eur J Dent. 2025 Mar 12;19(4):1084–91. doi: 10.1055/s-0045-1802345 (PMC12494432; doi:10.1055/s-0045-1802345)
Supplement: Supplementary file 1 — Supplementary Material [file 10-1055-s-0045-1802345-s2493780.pdf]

Supplementary Table S1 Impact of fasting on salivary cytokine levels before and after fasting across females and males

Variables	Median (IQR)		*p*-Value
	Male ($n = 22$)	Female ($n = 22$)	
IFN-γ before	2.22 (4.00)	3.65 (11.00)	0.188
IFN-γ after	3.96 (4.00)	3.67 (6.00)	0.944
IL-1β before	381.16 (651.00)	498.71 (702.00)	0.116
IL-1β after	521.36 (1,248.00)	682.91 (807.00)	0.827
IFN-α before	0.13 (0)	0.33 (0)	<0.001[a]
IFN-α after	0.39 (0)	0.30 (0)	0.054
TNF-α before	9.12 (13.00)	18.64 (21.00)	0.046[a]
TNF-α after	18.98 (18.00)	14.52 (22.00)	0.639
MCP-1 before	331.74 (480.00)	578.71 (763.00)	0.031[a]
MCP-1 after	517.00 (778.00)	467.89 (317.00)	0.707
IL-23 before	7.63 (8.00)	11.85 (16.00)	0.012[a]
IL-23 after	15.22 (6.00)	12.35 (16.00)	0.336
IL-33 before	1.70 (5.00)	5.30 (15.00)	0.004[a]
IL-33 after	10.80 (8.00)	7.70 (13.00)	0.082
IL-18 before	597.23 (1,529.00)	782.03 (794.00)	0.313
IL-18 after	967.41 (1,739.00)	1,065.90 (1,291.00)	0.778
IL-17 before	0.10 (0)	0.09 (0)	0.868
IL-17 after	0.10 (0)	0.09 (0)	0.475
IL-12 before	0.77 (1.00)	2.29 (7.00)	0.012[a]
IL-12 after	2.81 (3.00)	2.24 (4.00)	0.647
IL-10 before	1.63 (4.00)	4.68 (12.00)	0.005[a]
IL-10 after	4.63 (10.00)	5.23 (7.00)	0.869
IL-8 before	1,171.36 (1,616.00)	1,544.33 (1,235.00)	0.037[a]
IL-8 after	2,116.34 (2,466.00)	1,740.46 (1,284.00)	0.557
IL-6 before	4.83 (10.00)	9.57 (11.00)	0.039[a]
IL-6 after	12.43 (19.00)	8.52 (6.00)	0.496

Abbreviations: IFN, interferon; IL, interleukin; IQR, interquartile range; MCP, monocyte chemoattractant protein; TNF, tumor necrosis factor.
Note: The Mann–Whitney's *U* test was conducted to compare the median scores of dopamine and cytokines between males and females.
[a]Significant at 0.05.

 © 2025. The Author(s).
